# Thyroid Feedback Quantile-based Index correlates strongly to renal function in euthyroid individuals

**DOI:** 10.1080/07853890.2021.1993324

**Published:** 2021-11-02

**Authors:** Sijue Yang, Shuiqing Lai, Zixiao Wang, Aihua Liu, Wei Wang, Haixia Guan

**Affiliations:** a Department of Endocrinology and Metabolism, Institute of Endocrinology, NHC Key Laboratory of Diagnosis and Treatment of Thyroid Diseases, The First Affiliated Hospital of China Medical University, Shenyang, Liaoning, P.R. China†; b Department of Endocrinology, Guangdong Provincial People’s Hospital, Guangdong Academy of Medical Sciences, Guangzhou, Guangdong, P.R. China; c Department of Physical Examination Center, The First Hospital of China Medical University, Shenyang, Liaoning, P.R. China; d Department of Endocrinology and Metabolism, Peking University Third Hospital, Beijing, P.R. China; e The Second School of Clinical Medicine, Southern Medical University, Guangzhou, Guangdong, P.R. China

**Keywords:** Euthyroid individuals, sensitivity to thyroid hormone, thyroid feedback quantile-based index, renal function

## Abstract

**Background:**

Previous studies have reported a negative relationship between thyroid-stimulating hormone (TSH) and renal function in euthyroid individuals, but others have found that higher free thyroxine (FT_4_) was associated with an increased risk of chronic kidney disease. This study was designed to analyze the relationship between thyroid and renal function from a new perspective of sensitivity to thyroid hormone.

**Methods:**

This retrospective study included 2831 euthyroid individuals who underwent a health examination at the First Hospital of China Medical University between January 2017 and December 2018. Parametric Thyroid Feedback Quantile-based Index (PTFQI_FT4_), TSH index (TSHI), thyrotroph T_4_ resistance index (TT_4_RI), free triiodothyronine to FT_4_ ratio (FT_3_/FT_4_), the secretory capacity of the thyroid gland (SPINA-GT) and the sum activity of peripheral deiodinases (SPINA-GD) were calculated. We also innovated the TT_3_RI and PTFQI_FT3_ indices based on FT_3_ and TSH. Renal function was assessed by estimated glomerular filtration rate (eGFR) CKD-EPI and creatinine-cystatin C-KDIGO equations.

**Results:**

After adjustment of basic characteristics and comorbidities, linear regression showed that eGFR _CKD-EPI_ was positively associated with FT_3_/FT_4_ (*β* = 23.31), and inversely correlated to PTFQI _FT4_ (*β*= −2.69) (both *p* < .001). When comparing the fourth versus the first quartile of PTFQI _FT4_, the odds ratio (OR) for a reduced renal function was 1.89 (95% CI 1.28–2.80), and the OR was 0.64 (95% CI 0.43–0.95) when comparing quartiles of FT_3_/FT_4_ (both *p*_for trend_< .05). In addition, for every 1SD increase in PTFQI _FT4_, the OR for a reduced renal function was 1.27 (95%CI 1.10–1.47). TSHI, TT_4_RI and TT_3_RI also showed a negative correlation to renal function. Similar results were obtained in SPINA-GD as in FT_3_/FT_4_.

**Conclusions:**

In euthyroid individuals, decreased sensitivity to thyroid hormone is associated with reduced renal function. The composite PTFQI_FT4_ index correlates more strongly to renal function than TSH or T_4_ alone.

**KEY MESSAGES**
- Decreased sensitivity to thyroid hormone is associated with reduced renal function in the euthyroid population.
- The recently developed composite index PTFQI_FT4_ seems to correlate more strongly to renal function than individual TSH or FT_4_ parameters.
- Innovative indices TT_3_RI and PTFQI_FT3_ based on the interaction between T_3_ and TSH may also reflect sensitivity to thyroid hormone.

## Introduction

Impaired renal function and chronic kidney disease are global public health problems with growing prevalence and mortality, some of which can be attributed to hormonal and metabolic impact on renal function [1,2]. Thyroid hormones have been shown to affect glomerular filtration rate and renal tubular function [3]. There has been a large number of studies on the correlation between thyroid disorders and reduced renal function, but the results have been inconsistent [4–8]. Even in euthyroid population studies, no consensus has been reached on this topic. Some studies have shown that higher thyroid-stimulating hormone (TSH) levels within the reference range were associated with decreased estimated glomerular filtration rate (eGFR) or increased incidence of chronic kidney disease [9–14]. However, others have only reported a positive correlation between free triiodothyronine (FT_3_) and eGFR [15]. On the contrary, some studies have found that higher free thyroxine (FT_4_) was associated with decreased eGFR in the euthyroid population [16,17].

Classically, thyroid function is evaluated with simple serum FT_3_, FT_4_ and TSH levels. However, the complex interactions between FT_3_, FT_4_ and TSH can be assessed with sensitivity to thyroid hormone indices that can provide a new interpretation of thyroid status. In the central pituitary, thyroid hormones suppress TSH production by negative feedback, which can be assessed by TSH index (TSHI), thyrotroph T_4_ resistance index (TT_4_RI) and Parametric Thyroid Feedback Quantile-based Index (PTFQI) [18–20]. In the peripheral tissues, FT_3_ to FT_4_ ratio (FT_3_/FT_4_) and the sum activity of peripheral deiodinases (SPINA-GD) are indices reflecting the conversion efficiency of FT_4_ to FT_3_, which can indirectly estimate the bioavailability of thyroid hormones [21]. In the thyroid gland, its maximum secretory capacity can be calculated by SPINA-GT [21]. Recently, some researchers have utilized sensitivity to thyroid hormone indices in euthyroid populations to associate with obesity, metabolic syndrome, and diabetes [18,22], which are underlying diseases that could also lead to impaired renal function [23].

Whether sensitivity of thyroid hormone is associated with renal function remains unknown. Therefore, we investigated the relationship between thyroid and renal function from the perspective of sensitivity to thyroid hormone in the euthyroid population.

## Materials and methods

### Subjects

**Figure 1.**
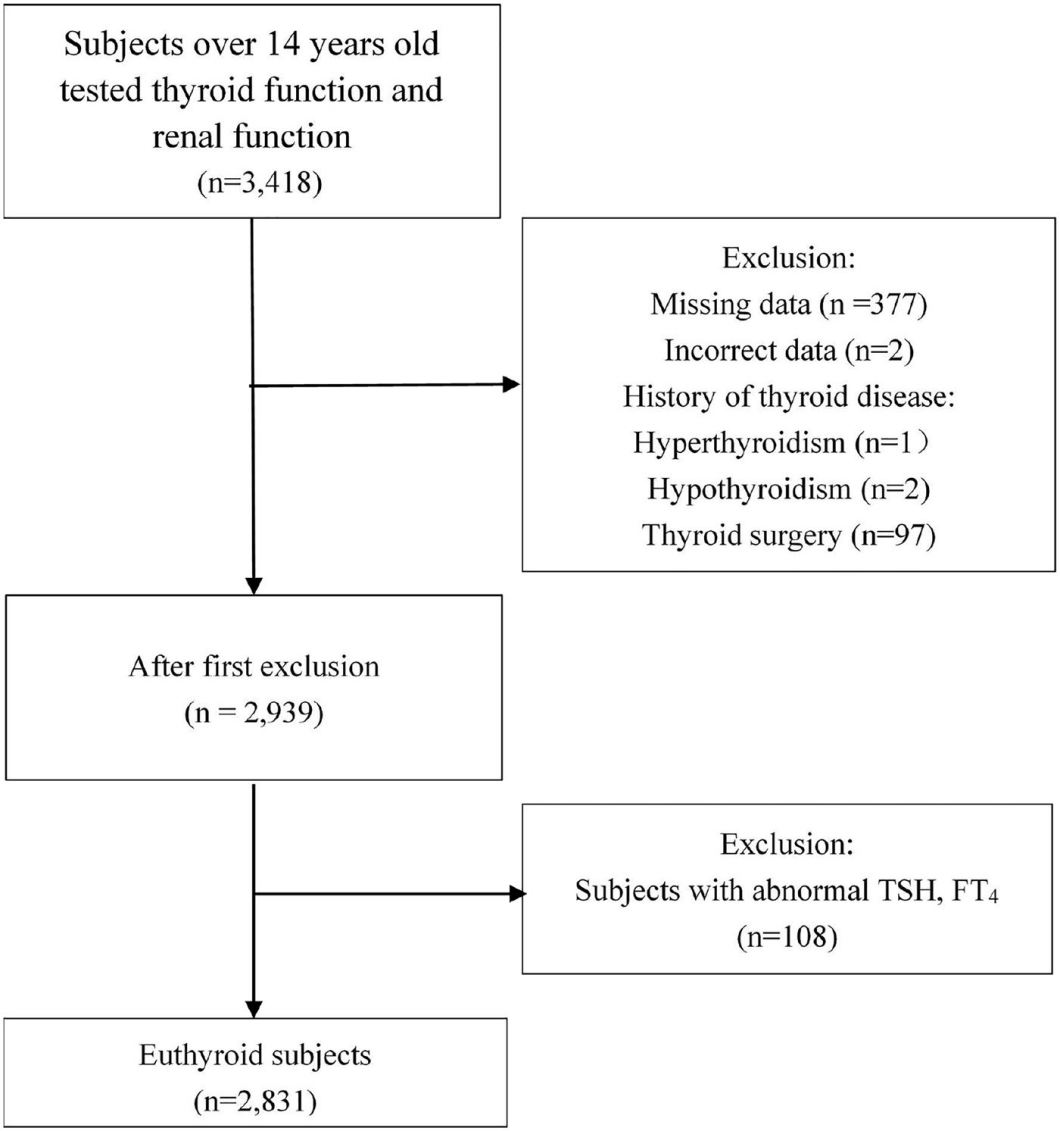
Flowchart of the inclusion and exclusion of participants. FT_4_: free thyroxine; TSH: thyroid-stimulating hormone.

All subjects were enrolled in an annual physical examination between January 2017 and December 2018. Data were retrospectively collected from the Physical Examination Centre of the First Hospital of China Medical University. We included subjects over 14 years old who had been tested for thyroid function, renal function, and other metabolism-related indicators, including fasting plasma glucose, lipid profiles and uric acid (*n* = 3,418). The exclusion criteria included: (1) missing records of medical history or measurements of anti-thyroid peroxidase antibody, anti-thyroglobulin antibody and anthropometric parameters such as height, weight, and waist circumference (*n* = 377); (2) incorrect data due to measurement or recording error (*n* = 2); (3) having a history of thyroid surgery, thyroid disorder (hyperthyroidism or hypothyroidism), or pituitary disorder (*n* = 100); and (4) subjects with abnormal TSH and/or FT_4_ (*n* = 108) (Figure 1). This retrospective study was approved by the Ethics Committee of the First Hospital of China Medical University. An informed consent waiver was obtained to use de-identified data [NO. (2019)241].

### Body measurements and laboratory examinations

The basic information including gender, age, height, weight, waist circumference, blood pressure and heart rate were measured and recorded. And the medical history was obtained from the inquiry. Body mass index was calculated as weight (kg) divided by height (m) squared. Venous blood samples were collected after an overnight fast. The automatic biochemical analyzer (Hitachi, Japan) was used to measure biochemical parameters, including serum creatinine, urea nitrogen, uric acid, cystatin C, fasting plasma glucose, haemoglobin A1c and lipid profiles. The subjects with a previous diagnosis of diabetes or measuring fasting plasma glucose ≥ 7.0 mmol/L or haemoglobin A1c ≥ 6.5% were defined as diabetic. Hypertension was defined as systolic blood pressure ≥140 mmHg and/or diastolic blood pressure ≥90 mmHg, or having a history of hypertension. Dyslipidemia was assessed by triglyceride ≥1.7 mmol/L or total cholesterol ≥5.2 mmol/L or high-density lipoprotein cholesterol ≤1.0 mmol/L or low-density lipoprotein cholesterol ≥3.4 mmol/L.

Serum levels of FT_3_, FT_4_, TSH, thyroid peroxidase antibody, and thyroglobulin antibody were measured by electrochemiluminescent immunoassays on Architect i2000SR (Abbott Laboratories, Chicago, IL, USA). Reference ranges for FT_4_, FT_3_, TSH, thyroid peroxidase antibody, and thyroglobulin antibody were 9.01–19.05 pmol/L, 2.63–5.70 pmol/L, 0.35–4.94 mIU/L, 0–5.61 IU/mL, 0–4.11 IU/mL, respectively. Euthyroid was defined as TSH and FT_4_ within reference ranges. Positive for thyroid peroxidase antibodies or thyroglobulin antibodies was defined as having a value above the upper limit.

Indices reflecting the sensitivity of thyroid hormone were calculated according to previous studies:

- FT_3_/FT_4_ ratio = FT_3_ (pmol/L)/FT_4_ (pmol/L). Higher FT_3_/FT_4_ indicates higher peripheral thyroid hormone activity.
- TSHI = ln TSH (mIU/L) + 0.1345 * FT_4_ (pmol/L) [19].
- TT_4_RI = FT_4_ (pmol/L) * TSH (mIU/L) [20]. For TSHI and TT_4_RI, the higher the values, the lower the central sensitivity to thyroid hormones.
- PTFQI_FT4_ is an approximation of the TFQI = cdfFT4−(1−cdfTSH) and can be adapted to different study populations. PTFQI_FT_4__ shows the difference between the FT_4_ and reversed TSH quantiles. PTFQI_FT4_ was calculated with the Excel spreadsheet formula: NORM.DIST (FT_4_-cell, μ FT_4_, σFT_4_, TRUE) + NORM.DIST [ln (TSH-cell), μ ln TSH, σln TSH, TRUE]-1 [18]. In our cohort, μ FT_4_, σFT_4_, μ ln TSH and σ ln TSH were 13.2678, 1.4871, 0.4757 and 0.4951, respectively. The value of PTFQI ranged from -1 to 1. The negative values indicated higher sensitivity to thyroid hormones in the pituitary; positive values indicated less sensitivity; the value of 0 indicated a normal sensitivity [18].
- Thyrotroph T_3_ resistance index (TT_3_RI) and PTFQI_FT3_: We innovated two new formulae based on the interaction between FT_3_ and TSH into the above formulae to obtain TT_3_RI and PTFQI_FT3_. The interpretation of TT_3_RI and PTFQI_FT3_ is similar to their respective original formula.
- Thyroid secretory capacity SPINA-GT = β_T_ (D_T_ + [TSH]) (1 + K_41_[TBG] + K_42_[TBPA]) [FT_4_]/α_T_[TSH].
- The sum activity of peripheral deiodinases SPINA-GD = β_31_ (K_M1_ + [FT_4_]) (1 + K_30_[TBG]) [FT_3_]/α_31_[FT_4_]. SPINA-GT and SPINA-GD parameters were calculated by the SPINA Thyr Software [21].

Renal function was assessed by eGFR using the Chronic Kidney Disease Epidemiology Collaboration (CKD-EPI) equation and creatinine-cystatin C-KDIGO (Cr–CysC) equation [24,25]. The number of patients in chronic kidney disease stages G3a to G5 was relatively small in the general physical examination population. Therefore, reduced renal function was defined as eGFR< 90 mL/min/1.73m^2^.

### Statistical analysis

Continuous variables were presented as mean ± SD or medians (interquartile ranges) for those with normal or skewed distributions, respectively. Categorical variables were shown as numbers (proportions). The *t*-tests and non-parametric Mann–Whitney tests were used to compare quantitative variables between the reduced renal function and normal renal function groups. Comparison of categorical variables was conducted with chi-square tests. The values of TT_4_RI, TT_3_RI, SPINA-GT and SPINA-GD were normalized by natural log (ln) transformation for skewed distributions, then multiple linear regression was used to analyze the relationship between thyroid parameters and eGFR. Multivariable logistic regression analysis was used to examine the association of thyroid parameters in quartiles with reduced renal function. We selected confounders based on the clinical relevance or univariate relationship (*p* < 0.2) with renal function and sensitivity to thyroid hormone. Model 1 adjusted age and sex. Model 2 further adjusted for body mass index, waist circumference, heart rate, diabetes, hypertension, dyslipidemia, thyroid peroxidase antibody positivity and thyroglobulin antibody positivity. Analyses were also performed based on age (younger or older than 65 years) and renal function (normal or reduced renal function). In addition, we performed sensitivity analyses in non-diabetic and non-hypertensive subjects, respectively. All calculated *p-*values were two-sided, and a *p-*value< .05 was considered statistically significant. All analyses were conducted with the SPSS 22.0 software.

## Results

### Clinical characteristics of the participants

A total of 2831 euthyroid participants were included in this study, with a mean age of 51.08 years (SD 10.33 years, min 16 years, max 86 years); among them, 1717 (60.65%) were men. The clinical characteristics were shown in Table 1. The overall prevalence of reduced renal function based on eGFR_CKD-EPI_ was 10.21% (289/2831). Participants who had lower eGFR_CKD-EPI_ were older, more likely to be male, and have diabetes or hypertension (all *p* < .05). In addition, participants with lower eGFR_CKD-EPI_ had higher body mass index, waist circumference, and serum uric acid levels (all *p* < .05). They also had lower FT_3_ levels, FT_3_/FT_4_ ratio and SPINA-GD (all *p* < .05). Although the indices reflecting central sensitivity of thyroid hormone were higher and the SPINA-GT was lower in patients with lower eGFR _CKD-EPI_, there was no statistically significant difference compared to the higher eGFR_CKD-EPI_ group.

**Table 1.** Clinical characteristics of all participants.

	Normal renal function eGFR_CKD-EP_*_I_*≥ 90 mL/min/1.73m^2^	Reduced renal function eGFR_CKD-EP_*_I_* <90 mL/min/1.73m^2^	*p*
*N*	2542	289	
Age (years)	49.79 ± 9.51	62.38 ± 10.41	<.001
Men (*n*, %)	1510 (59.40)	207 (71.63)	<.001
BMI (Kg/m^2^)	25.55 ± 3.43	25.98 ± 3.18	.04
WC (cm)	86.80 ± 10.17	89.30 ± 9.70	<.001
Heart rate (bpm)	74.64 ± 10.92	73.21 ± 11.13	.04
Uric acid (umol/L)	328.62 ± 87.48	362.05 ± 91.74	<.001
Diabetes (*n*, %)	321 (12.63)	58 (20.07)	<.001
Hypertension (*n*, %)	962 (37.84)	165 (57.09)	<.001
Dyslipidemia (*n*, %)	1694 (66.64)	204 (70.59)	.18
FT_3_ (pmol/L)	4.37 ± 0.53	4.26 ± 0.48	.001
FT_4_ (pmol/L)	13.26 ± 1.49	13.36 ± 1.44	.27
TSH (mIU/L)	1.62 (1.05)	1.68 (1.21)	.27
FT_3_/FT_4_	0.33 ± 0.05	0.32 ± 0.04	<.001
TSHI	2.26 ± 0.50	2.30 ± 0.53	.15
TT_4_RI	21.36 (14.09)	22.27 (16.74)	.19
PTFQI_FT4_	–0.01 ± 0.38	0.04 ± 0.38	.07
TT_3_RI	7.04 (4.60)	6.91 (5.10)	.85
PTFQI_FT3_	0.01 ± 0.38	–0.03 ± 0.38	.16
SPINA-GT (pmol/s)	2.72 (1.25)	2.66 (1.34)	.53
SPINA-GD (nmol/s)	30.53 (5.81)	29.65 (5.45)	.001
TPOAb positive (*n*, %)	304 (11.96)	42 (14.53)	.21
TgAb positive (*n*, %)	508 (19.98)	56 (19.38)	.81
Urea (mmol/L)	5.13 ± 1.24	6.37 ± 2.29	<.001
Serum creatinine (umol/L)	61.79 ± 12.00	88.09 ± 54.41	<.001
Cystatin-C (mg/L)	0.73 ± 0.13	1.01 ± 0.39	<.001
eGFR _CKD–EPI_ (mL/min/1.73 m^2^)	106.83 ± 9.27	80.57 ± 12.46	<.001
eGFR _Cr-CysC_ (mL/min/1.73m^2^)	111.81 ± 13.08	81.16 ± 15.48	<.001

Data are means ± standard deviations or medians (interquartile ranges) for continuous variables, and numbers (proportions) for categorical variables.

BMI: body mass index; WC: waist circumference; FT_3_: free triiodothyronine; FT_4_: free thyroxine; TSH: thyroid-stimulating hormone; FT_3_/FT_4_, FT_3_ to FT_4_ ratio, TSHI: TSH index; TT_4_RI: thyrotroph T_4_ resistance index; PTFQI_FT4_: Parametric Thyroid Feedback Quantile-based Index calculated by FT_4_; PTFQI_FT3_: Parametric Thyroid Feedback Quantile-based Index calculated by FT_3_; TT_3_RI: thyrotroph T_3_ resistance index; SPINA-GT: the secretory capacity of the thyroid gland; SPINA-GD: the sum activity of peripheral deiodinases; TPOAb: thyroid peroxidase antibody; TgAb: thyroglobulin antibody; eGFR_CKD-EPI_: estimated glomerular filtration rate based on CKD-EPI equation; eGFR_Cr-CysC_: eGFR based on serum creatinine and cystatin-C.

### Association of sensitivity of thyroid hormone indices with eGFR

Linear regression analysis was used to examine the relationship between thyroid parameters and eGFR. In Model 1, after adjustment of age and sex, eGFR_CKD-EPI_ was positively associated with the level of FT_3_/FT_4_ (*β* = 20.60, *p* < .001) and negatively associated with PTFQI_FT4_ (*β* = −2.60, *p* < .001). The other indices reflecting central sensitivity of thyroid hormone (TSHI, Ln TT_3_RI, Ln TT_4_RI, except for PTFQI_FT3_) were also inversely correlated to eGFR_CKD-EPI_ (*β* = −1.90 to −1.22, all *p* < .001). Ln SPINA-GT and Ln SPINA-GD were positively associated with eGFR _CKD-EPI_ (*β* = 1.50 and 6.64, respectively, both *p* < .01). The above associations remained significant after further adjustment in Model 2. All correlations remained consistent when analyzed with eGFR_Cr-CysC_, except for the association between Ln SPINA-GT and eGFR_Cr-CysC_ (Table 2, Supplementary Figure 1).

**Table 2.** Association of sensitivity of thyroid hormone indices with eGFR by linear regression.

	eGFR_CKD-EPI_			eGFR_Cr-CysC_		
	*β*	95%CI	*p*	*β*	95%CI	*p*
Model 1						
FT_3_/FT_4_	20.60	13.68 ∼ 27.52	<.001	18.27	8.93 ∼ 27.60	<.001
TSHI	–1.90	–2.56∼ −1.25	<.001	–2.65	–3.54∼−1.77	<.001
Ln TT_4_RI	–1.85	–2.53∼−1.17	<.001	–2.47	–3.38∼−1.56	<.001
PTFQI_FT4_	–2.60	–3.47∼−1.72	<.001	–3.96	–5.14∼−2.79	<.001
Ln TT_3_RI	–1.22	–1.88∼−0.55	<.001	–1.85	–2.74∼−0.96	<.001
PTFQI_FT3_	–0.27	–1.14 ∼ 0.61	.55	–2.07	–3.25∼−0.90	.001
Ln SPINA-GT	1.50	0.55 ∼ 2.46	.002	1.47	0.18 ∼ 2.76	.03
Ln SPINA-GD	6.64	4.34 ∼ 8.95	<.001	6.08	2.97 ∼ 9.19	<.001
Model 2						
FT_3_/FT_4_	23.31	16.34 ∼ 30.28	<.001	23.77	14.45 ∼ 33.09	<.001
TSHI	–1.92	–2.58∼−1.26	<.001	–2.52	–3.40∼−1.64	<.001
Ln TT_4_RI	–1.85	–2.53∼−1.17	<.001	–2.29	–3.20∼−1.38	<.001
PTFQI_FT4_	–2.69	–3.57∼−1.81	<.001	–3.94	–5.10∼−2.77	<.001
Ln TT_3_RI	–1.15	–1.82∼−0.48	.001	–1.54	–2.43∼−0.65	.001
PTFQI_FT3_	–0.06	–0.94 ∼ 0.82	.90	–1.44	–2.62∼−0.26	.02
Ln SPINA-GT	1.41	0.44 ∼ 2.37	.004	1.09	–0.19 ∼ 2.38	.10
Ln SPINA-GD	7.56	5.24 ∼ 9.88	<.001	7.94	4.83 ∼ 11.04	<.001

TT_4_RI, TT_3_RI, SPINA-GT and SPINA-GD were ln-transformed for normal distribution before linear regression analysis.

Model 1: age and sex were adjusted.

Model 2: body mass index, waist circumference, heart rate, diabetes, hypertension, dyslipidemia, TPOAb positive and TgAb positive were further adjusted based on model 1.

FT_3_: free triiodothyronine; FT_4_: free thyroxine; TSH: thyroid-stimulating hormone; FT_3_/FT_4_, FT_3_ to FT_4_ ratio, TSHI: TSH index; TT_4_RI: thyrotroph T_4_ resistance index; PTFQI_FT4_: Parametric Thyroid Feedback Quantile-based Index calculated by FT_4_; PTFQI_FT3_: Parametric Thyroid Feedback Quantile-based Index calculated by FT_3_; TT_3_RI: thyrotroph T_3_ resistance index; SPINA-GT: the secretory capacity of the thyroid gland; SPINA-GD: the sum activity of peripheral deiodinases; TPOAb: thyroid peroxidase antibody; TgAb: thyroglobulin antibody; eGFR _CKD-EPI_: estimated glomerular filtration rate based on CKD-EPI equation; eGFR_Cr-CysC_: eGFR based on serum creatinine and cystatin-C.

### Association of sensitivity of thyroid hormone indices with reduced renal function

**Figure 2.**
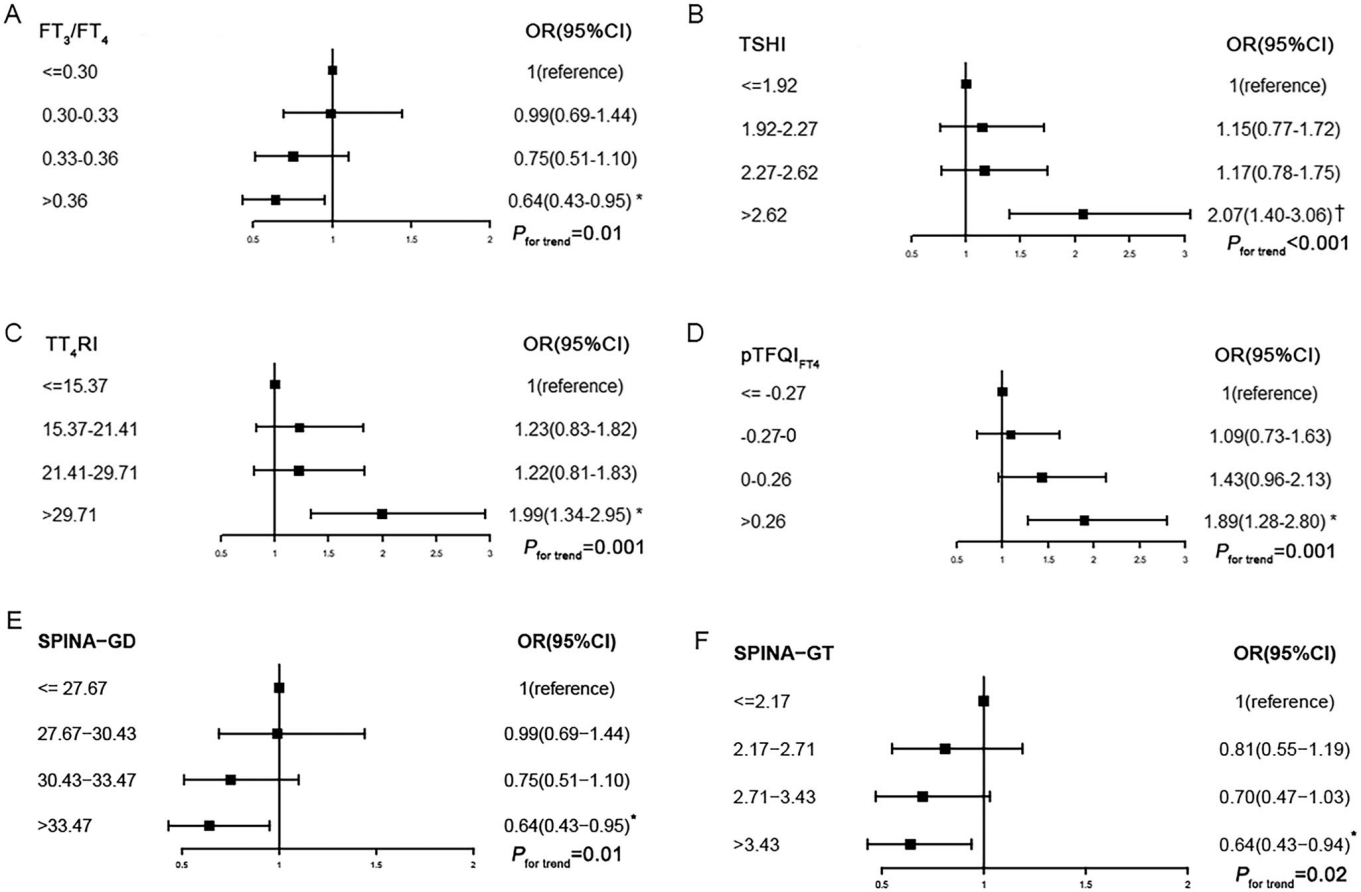
Association of sensitivity of thyroid hormone indices quartiles with reduced renal function. *P*_for trend_ calculated with sensitivity to thyroid hormone indices quartile ordinal as a continuous variable. Model 2: age, sex, body mass index, waist circumference, heart rate, diabetes, hypertension, dyslipidemia, TPOAb positive and TgAb positive were adjusted. FT_3_/FT_4_: free triiodothyronine to free thyroxine ratio; TSHI: thyroid-stimulating hormone index; TT_4_RI: thyrotroph T_4_ resistance index; PTFQI_FT4_: Parametric Thyroid Feedback Quantile-based Index calculated by FT_4_; SPINA-GT: the calculated secretory capacity of the thyroid gland; SPINA-GD: the sum activity of peripheral deiodinases; TPOAb: thyroid peroxidase antibody; TgAb: thyroglobulin antibody. *p<.05; ^†^*p*<.001.

When thyroid parameters are categorized into quartiles, the odds ratio (OR) of the fourth versus the first quartile of PTFQI_FT4_ was 1.89 (95%CI 1.28–2.80, *p*_for trend_ = .001) for reduced renal function (eGFR_CKD-EPI_ <90 mL/min/1.73m^2^). The ORs of the fourth versus the first quartile of TSHI and TT_4_RI for reduced renal function were 2.07 (95%CI 1.40–3.06) and 1.99 (95%CI 1.34–2.95), respectively (both *p*_for trend_ < .05). The risk of reduced renal function in the highest quartile of FT_3_/FT_4_ was significantly lower than in the lowest quartile (OR = 0.64, 95%CI 0.43–0.95, *p*_for trend_ = .01). The results of SPINA-GT and SPINA-GD were similar to that of FT_3_/FT_4_ (Figure 2).

Based on Model 2 logistic regression, for every 1SD increase in FT_3_/FT_4_ or PTFQI_FT4_, the ORs for reduced renal function (eGFR_CKD-EPI_ <90 mL/min/1.73m^2^) were 0.80 (95%CI 0.69–0.93) and 1.27 (95%CI 1.10–1.47), respectively. TSHI, TT_4_RI and TT_3_RI showed similar results as PTFQI_FT4_. The OR for reduced renal function with every 1SD increase in SPINA-GD was similar to FT_3_/FT_4_. However, there was no significant relationship between SPINA-GT and reduced renal function. The OR for a reduced renal function was 1.27 (95%CI 1.11–1.45) with every 1SD increase in TSH. PTFQI_FT3_ did not show a relationship with reduced renal function. These results remained consistent when renal function was assessed based on the eGFR_Cr-CysC_ equation. However, regression models with FT_3_ and FT_4_ were inconsistent when using different eGFR equations (Table 3).

**Table 3.** Association of sensitivity of thyroid hormone indices with reduced renal function by logistic regression.

	Reduced renal function (eGF*R* < 90 mL/min/1.73m^2^)					
	eGFR_CKD-EPI_			eGFR_Cr-CysC_		
(+1SD)	OR	95%CI	*p*	OR	95%CI	*p*
Model 1						
FT_3_	0.84	0.72 ∼ 0.97	.02	0.92	0.80 ∼ 1.05	.22
FT_4_	1.07	0.93 ∼ 1.22	.33	1.13	0.99 ∼ 1.29	.07
TSH	1.29	1.13 ∼ 1.47	<.001	1.25	1.10 ∼ 1.42	.001
FT_3_/FT_4_	0.82	0.71 ∼ 0.95	.007	0.85	0.74 ∼ 0.98	.02
TSHI	1.30	1.13 ∼ 1.50	<.001	1.26	1.10 ∼ 1.45	.001
TT_4_RI	1.32	1.15 ∼ 1.51	<.001	1.29	1.13 ∼ 1.47	<.001
PTFQI_FT4_	1.27	1.10 ∼ 1.46	.001	1.24	1.09 ∼ 1.43	.002
TT_3_RI	1.22	1.07 ∼ 1.39	.003	1.21	1.07 ∼ 1.38	.003
PTFQI_FT3_	1.08	0.94 ∼ 1.23	.31	1.08	0.95 ∼ 1.24	.25
SPINA-GT	1.15	0.75 ∼ 1.00	.05	0.92	0.81 ∼ 1.05	.24
SPINA-GD	0.82	0.71 ∼ 0.95	.007	0.85	0.74 ∼ 0.98	.02
Model 2						
FT_3_	0.83	0.71 ∼ 0.97	.02	0.90	0.78 ∼ 1.04	.16
FT_4_	1.09	0.95 ∼ 1.25	.22	1.15	1.00 ∼ 1.31	.05
TSH	1.27	1.11 ∼ 1.45	.001	1.22	1.07 ∼ 1.39	.003
FT_3_/FT_4_	0.80	0.69 ∼ 0.93	.003	0.83	0.72 ∼ 0.96	.01
TSHI	1.28	1.11 ∼ 1.48	.001	1.24	1.08 ∼ 1.42	.003
TT_4_RI	1.30	1.14 ∼ 1.49	<.001	1.26	1.11 ∼ 1.44	.001
PTFQI_FT4_	1.27	1.10 ∼ 1.47	.001	1.24	1.07 ∼ 1.42	.003
TT_3_RI	1.19	1.04 ∼ 1.36	.01	1.18	1.04 ∼ 1.34	.01
PTFQI_FT3_	1.05	0.92 ∼ 1.21	.48	1.05	0.92 ∼ 1.20	.50
SPINA-GT	0.88	0.77 ∼ 1.02	.08	0.94	0.82 ∼ 1.07	.37
SPINA-GD	0.80	0.69 ∼ 0.93	.003	0.83	0.72 ∼ 0.96	.01

ORs are estimated with generalized logistic regression models for the increase of thyroid parameters (1 SD).

Model 1: age and sex were adjusted.

Model 2: body mass index, waist circumference, heart rate, diabetes, hypertension, dyslipidemia, TPOAb positive and TgAb positive were further adjusted based on model 1.

FT_3_: free triiodothyronine; FT_4_: free thyroxine; TSH: thyroid-stimulating hormone; FT_3_/FT_4_, FT_3_ to FT_4_ ratio, TSHI: TSH index; TT_4_RI: thyrotroph T_4_ resistance index; PTFQI_FT4_: Parametric Thyroid Feedback Quantile-based Index calculated by FT_4_; PTFQI_FT3_: Parametric Thyroid Feedback Quantile-based Index calculated by FT_3_; TT_3_RI: thyrotroph T_3_ resistance index; SPINA-GT: the secretory capacity of the thyroid gland; SPINA-GD: the sum activity of peripheral deiodinases; TPOAb: thyroid peroxidase antibody; TgAb: thyroglobulin antibody; eGFR_CKD-EPI_: estimated glomerular filtration rate based on CKD-EPI equation; eGFR_Cr-CysC_: eGFR based on serum creatinine and cystatin-C.

We also performed stratified analyses in different age groups. The relationship between the sensitivity of thyroid hormone indices with eGFR and the effect of every 1SD increase in these indices on reduced renal function persisted in the younger group. However, we did not obtain significant results in the subjects aged over 65 years. The age strata only showed significant interactions in the relationships of TSHI, TT_3_RI and SPINA-GT with eGFR_CKD-EPI_ decline (*p*_for interaction_= 0.04, 0.03, and 0.01, respectively) (Supplementary Tables 1 and 2). Additionally, the indices reflecting sensitivity to thyroid hormone showed a significant association with eGFR in subjects with normal renal function, but there was no significance in subjects with reduced renal function (Supplementary Table 3). Furthermore, to examine the possible effects of diabetes or hypertension on renal function, sensitivity analyses were performed in non-diabetic and non-hypertensive subjects, respectively. The association between the sensitivity of thyroid hormone indices with eGFR and reduced renal function persisted within the non-diabetic population. The association in non-hypertensive subjects became non-significant for some indices, although the magnitudes for the ORs were close to the main sample (Supplementary Tables 4 and 5).

## Discussion

In this population-based study, we found a relationship between indices measuring sensitivity to thyroid hormone and eGFR or the risk of reduced renal function in the euthyroid Chinese population. This correlation was most significant in subjects aged ≤65 years old, without diabetes or hypertension, or with normal renal function. In addition, we put forward the innovative TT_3_RI and PTFQI_FT3_ according to the established formulae and found that there was a relationship between TT_3_RI and renal function. These composite indices detected a stronger correlation between thyroid and renal dysfunction and provided a new thought for assessing thyroid function in the euthyroid population.

Given the effect of ageing, diabetes, and hypertension on renal function, we analyzed these specific subgroups. The relationships that we detected in the overall cohort were also observed in non-diabetic subjects and subjects ≤65 years old or with normal renal function. However, we failed to detect any significant correlation in the older group, which may be due to the insufficient sample size (*n* = 230) and the need for a lower threshold of eGFR to define a reduced renal function in the elderly. The relationship between these composite indices and reduced renal function in the subjects without hypertension was very close to the entire cohort, but the *p-*values were only marginally significant. The reduced renal function group was relatively small (*n* = 289 and 315 based on eGFR_CKD-EPI_ and eGFR_Cr-CysC_, respectively), which may have contributed to the lack of statistical significance.

The correlation between thyroid function and renal function remains a relevant topic as a consensus has not been reached despite much discussion in the literature from the past five years. Our results on the negative relationship between TSH and renal function were consistent with several previous findings [9,11,14]. This observation is also supported by a study on urine albumin excretion that found higher TSH (even in the euthyroid range) was associated with a higher risk of microalbuminuria [10]. Furthermore, in line with a Dutch study and a Mendelian randomization study, we did not find a significant relationship between FT_4_ and renal function [13,15]. Similar to the Dutch study, we are one of the few studies that have measured FT_3_, and both studies suggest a protective effect of FT_3_ on renal function in the euthyroid population [15]. However, a Korean study reported that increased FT_4_ was associated with decreased eGFR, and the authors attributed it to functional hypothyroidism at the tissue level [17]. In support of the Korean study is a Chinese study that did not find a significant relationship between FT_3_ and renal function in euthyroid participants, but observed that the risk of chronic kidney disease was 1.763-fold higher in the highest quartiles of FT_4_ compared with the lowest quartile [16]. The race, age, and confounding factors adjustments in the Chinese cross-sectional study were similar to those in our study. All in all, these inconsistent results suggest that the dynamics of thyroid hormones with renal dysfunction in the euthyroid population require a new explanation.

The secretion of thyroid hormone is regulated by hypothalamic-pituitary-thyroid (HPT) axis. Thyrotropin-releasing hormone (TRH) from the hypothalamus promotes the synthesis and release of TSH from the anterior pituitary, which plays an important role in all stages related to the production and secretion of thyroid hormones from the thyroid gland. The levels of TRH and TSH are in turn modulated by the negative feedback of thyroid hormones. Thyroid hormones are mainly secreted in the form of T_4_, which is catalyzed by deiodinases to form the bioactive T_3_. Both will bind to carrier proteins in the circulation and enter cells *via* membrane transporters. T_3_ then further binds to the nuclear thyroid hormone receptors. Therefore, thyroid function is regulated by the HPT axis and other factors associated with thyroid hormone conversion and bioactivity [26].

Given the complex interactions within the HPT axis, a single parameter may not reflect true thyroid status. Initial studies proposed TSHI and TT_4_RI to assess the central sensitivity of thyroid hormone [19,20]. The latest studies reported that obesity-related reduced sensitivity to thyroid hormone can be reversed after bariatric surgery-induced weight loss [27]. Among type 2 diabetes patients, elevated TSHI and TT_4_RI were associated with an increased prevalence of kidney disorders [28]. In 2019, Laclaustra et al. proposed a new index, PTFQI, that was thought to be more stable than TSHI and TT_4_RI. Their study showed the association of reduced sensitivity to thyroid hormone with obesity, metabolic syndrome, and diabetes even in the euthyroid population. They recommended that the new index can be used to identify reduced sensitivity to thyroid hormone [18]. Recently, these indices were assessed in a Chinese euthyroid cohort study, which concluded that thyroid hormone sensitivity was associated with adipocyte fatty acid-binding proteins [22].

Due to the inconsistencies in studies that employed simplified thyroid hormone measurements and the complex interactions in the HPT axis, we set out to explore the unsolved debate of the relationship between thyroid and renal function within the perspective of thyroid hormone sensitivity by using composite indices. Hence, in our study, the relationship between the thyroid and renal function was more stable when using composite sensitivity of thyroid hormone indices. Unlike the singular thyroid function parameters, we found consistent relationships between composite indices and reduced renal function, even when using different eGFR equations. In addition, these correlations were slightly stronger when using composite indices rather than using individual thyroid hormone parameters. For example, the OR for a reduced renal function was 1.30 versus 1.27 with every 1SD increase in TT_4_RI and TSH, respectively. Furthermore, we observed reduced values of FT_3_/FT_4_ and SPINA-GD in subjects with reduced renal function. These indices that contain FT_3_ can better reflect the peripheral action of thyroid hormones because the bioactive FT_3_ is converted from FT_4_ by deiodinases in the peripheral tissues and has a much higher affinity with thyroid receptors than T_4_. Therefore, reduced sensitivity to thyroid hormone at the level of deiodinases might be present in subjects with reduced renal function. Unfortunately, most of the previous studies lacked FT_3_ measurements, and hence the existing composite indices are all based on FT_4_. With the advantage of measuring FT_3_, we tried substituting FT_3_ into the existing formulae to obtain new indices, and excitingly, we obtained strong correlations. We hope that this pioneering attempt could prompt more detailed research in utilizing FT_3_.

In a previous study, a higher value of PTFQI indicated higher TSH than that expected for the actual FT_4_, indicating a lower sensitivity to FT_4_ [18]. However, the reduced suppression of TSH may not only present a reduced sensitivity to thyroid hormone but also reflect an increased set-point of homeostasis, which is commonly observed in type 2 allostasis. Allostasis is defined as a dynamic response to maintain stability and consists of two types of allostatic load. Long-term type 2 allostatic load can contribute to obesity, hypertension, type 2 diabetes, and dyslipidemia [29]. The metabolic disorders caused by type 2 allostatic load have a close connection with renal dysfunction. Therefore, reduced renal function may be due to type 2 allostasis, and metabolic diseases serve as intermediaries. Although we have adjusted for potential metabolic variables, we cannot completely rule out the lifestyle-related confounders. But we are assured of the results when the same conclusions were obtained in the subgroup analysis that excluded participants with diabetes or hypertension. Moreover, the production of TSH and thyroid hormones tend to be upregulated in type 2 allostasis [29]. However, the subjects included in this study all had normal TSH and FT_4_ levels. Therefore, we believe that the elevated PTFQI in subjects with renal dysfunction mainly reflects reduced central sensitivity of thyroid hormone, but also, to a lesser extent, an increased set-point caused by type 2 allostatic load.

Past publications have evaluated in detail the potential mechanisms related to the connection between thyroid hormones and renal function. Thyroid hormones influence renal function by exerting effects on the following aspects: 1) system hemodynamics, including cardiac and vascular function, renin-angiotensin system and blood volume; 2) glomerular architecture, renal vasculature and the permeability of glomerular capillary; and 3) tubular function, for example, sodium and water homeostasis [30]. The mechanisms underlying the association between sensitivity to thyroid hormone and renal function remain to be further explored. But the relative insufficiency of thyroid hormones caused by decreased thyroid hormone sensitivity might also affect renal function through the above mechanisms. Furthermore, TSH receptors are expressed in many tissues other than the thyroid, including the kidneys [31]. This suggests that TSH may influence renal function by directly acting on renal cells or *via* indirect effects on systems other than the thyroid.

Renal dysfunction, in turn, interferes with the HPT axis and peripheral hormone metabolism. Previous studies have demonstrated that end-stage renal disease might alter TSH levels by dampening pituitary response to TRH, interfering with TSH diurnal rhythm and glycosylation, and reducing TSH clearance rate [32]. Uraemic toxins and malnutrition also affect peripheral hormone metabolism by impairing the binding of T_4_ with carrier proteins, inhibiting cellular uptake of T_4_, and reducing the peripheral conversion from T_4_ to T_3_ [32]. The previously detected positive relationship between creatinine clearance and SPINA-GT also points to the impairment of uraemic toxins on thyroid activity [33]. And various conditions caused by kidney diseases (such as metabolic acidosis, trace element deficiencies, impaired clearance and retention of iodine, and vitamin D deficiency) and medications used in such patients are risk factors for thyroid dysfunction [34,35]. In addition, chronic kidney disease is a common pattern of non-thyroidal illness syndrome, which is characterized as decreased free or total T_3_ and impaired carrier protein binding to thyroid hormones. In severe cases, the set-point of the HPT axis is downregulated. TSH is reduced in this condition, even though the level of T_4_ is normal or decreased [29,36]. Others have also detected that a decreased blood urea after a period of low protein diet could improve the low T_3_ syndrome in renal dysfunction patients [33]. This finding also suggests a negative effect of azotaemia on thyroid function.

Though this study is novel and has obtained consistently significant results between different indices and formulae, there are also some limitations. The first is the lack of data on medication and 2-hour post-load serum glucose levels in this study. Thus, the influence of residual confounding factors must be considered. In addition, reduced renal function outcome was based on measurements at a single time point. Therefore, we used both the eGFR_CKD-EPI_ equation and the eGFR_Cr-CysC_ equation to mitigate this limitation and have obtained consistent results with either equation. Both of these equations have been previously confirmed to be suitable for the evaluation of the glomerular filtration rate in the Chinese population [24]. Moreover, we defined reduced renal function as eGFR <90mL/min/1.73m^2^, which is useful for early detection and management of renal dysfunction. Lastly, due to the limitations of a cross-sectional study design, we failed to obtain causative association. Hence, more large longitudinal studies are needed to clarify the causal relationship.

In conclusion, we have found that reduced renal function was associated with decreased central sensitivity and reduced peripheral activity of thyroid hormones in the euthyroid population, particularly in those aged 65 years or under, without diabetes, or with normal renal function. Compared with individual parameters such as TSH or FT_4_, composite indices such as TFQI provided a more systemic and comprehensive way to evaluate thyroid homeostasis. And it was possible to create composite indices, such as TT_3_RI and PTFQI_FT3_, based on the interactions between FT_3_ and TSH. Future large prospective studies are needed to confirm these findings and more attention should be paid to the epidemiologic and mechanistic research in thyroid hormone sensitivity.

## Supplementary Material

Supplemental Material

## Data Availability

Due to the nature of this research, participants of this study did not agree for their data to be shared publicly, so supporting data is not available.
