# Supplementary material for: Thyroid Feedback Quantile-based Index correlates strongly to renal function in euthyroid individuals
Source: Ann Med. 2021 Nov 2;53(1):1945–55. doi: 10.1080/07853890.2021.1993324 (PMC8567884; doi:10.1080/07853890.2021.1993324)
Supplement: Supplemental Material [file IANN_A_1993324_SM4926.docx]

**Supplementary Data**

**Thyroid Feedback Quantile-Based Index Correlates Strongly to Renal Function in Euthyroid Individuals**

Sijue Yang, MBBS^1*^, Shuiqing Lai, M.D.^2*^, Zixiao Wang, M.D. and Ph.D.^3^, Aihua Liu, M.D. and Ph.D.^4^, Wei Wang, M.D.^3^, Haixia Guan, M.D. and Ph.D.^2, 5^

^1^ Department of Endocrinology and Metabolism, Institute of Endocrinology, NHC Key Laboratory of Diagnosis and Treatment of Thyroid Diseases, The First Affiliated Hospital of China Medical University, 155 Nanjing Bei Street, Shenyang, Liaoning 110001, P.R. China^[[1]](#footnote-1)^. ^2^ Department of Endocrinology, Guangdong Provincial People's Hospital, Guangdong Academy of Medical Sciences, 106 Zhongshan Er Road, Guangzhou, Guangdong 510080, P.R. China. ^3^ Department of Physical Examination Center, The First Hospital of China Medical University, 155 Nanjing Bei Street, Shenyang, Liaoning 110001, P.R. China. ^4^ Department of Endocrinology and Metabolism, Peking University Third Hospital, Beijing 100191, P.R. China. ^5^ The Second School of Clinical Medicine, Southern Medical University, Guangzhou, Guangdong 510000, P.R. China

***Sijue Yang and Shuiqing Lai contributed equally to this work.**

**Corresponding author:**

Haixia Guan, M.D. and Ph.D., Email: [hxguan@vip.126.com](mailto:hxguan@vip.126.com)

Department of Endocrinology, Guangdong Provincial People's Hospital, Guangdong Academy of Medical Sciences, 106 Zhongshan Er Road, Guangzhou, Guangdong 510080, P.R. China

The Second School of Clinical Medicine, Southern Medical University, Guangzhou, Guangdong 510000, P.R. China

Telephone: 86-18602491031

**Co-corresponding author:**

Wei Wang, MD, Email: 6899wangwei@163.com

Department of Physical Examination Center, The First Hospital of China Medical University, Shenyang 110001, China

**Supplementary table 1. Association of sensitivity of thyroid hormone indices with eGFR by linear regression stratified by age**

|  | **eGFR _CKD-EPI_** | | | **eGFR _Cr-CysC_** | | |
| --- | --- | --- | --- | --- | --- | --- |
|  | **β** | **95%CI** | ***P*** | **β** | **95%CI** | ***P*** |
| **Younger than 65 years** | | | | | | |
| FT_3_/FT_4_ | 22.90 | 15.85~22.96 | <0.001 | 22.10 | 12.61~31.59 | <0.001 |
| TSHI | -2.08 | -2.75~ -1.41 | <0.001 | -2.65 | -3.56~ -1.75 | <0.001 |
| Ln TT_4_RI | -2.02 | -2.72~ -1.33 | <0.001 | -2.45 | -3.38~ -1.51 | <0.001 |
| PTFQI_FT4_ | -2.88 | -3.76~ -1.99 | <0.001 | -4.04 | -5.22~ -2.85 | <0.001 |
| Ln TT_3_RI | -1.32 | -2.00~ -0.63 | <0.001 | -1.73 | -2.65~ -0.81 | <0.001 |
| PTFQI_FT3_ | -0.29 | -1.19~ 0.61 | 0.52 | -1.75 | -2.96~ -0.55 | 0.004 |
| Ln SPINA-GT | 1.67 | 0.67~ 2.67 | 0.001 | 1.38 | 0.05~ 2.72 | 0.04 |
| Ln SPINA-GD | 7.44 | 5.08~ 9.80 | <0.001 | 7.38 | 4.21~ 10.56 | <0.001 |
| **Older than 65 years** | | | | | | |
| FT_3_/FT_4_ | 24.76 | -8.81~58.32 | 0.15 | 34.22 | -8.08~76.53 | 0.11 |
| TSHI | -0.32 | -3.12~ 2.48 | 0.82 | -1.33 | -4.85~ 2.20 | 0.46 |
| Ln TT_4_RI | -0.26 | -3.06~ 2.55 | 0.86 | -1.07 | -4.60~ 2.47 | 0.55 |
| PTFQI_FT4_ | 0.07 | -4.13~ 4.28 | 0.97 | -2.07 | -7.37~ 3.22 | 0.44 |
| Ln TT_3_RI | 0.25 | -2.46~ 2.96 | 0.85 | -0.30 | -3.72~ 3.13 | 0.86 |
| PTFQI_FT3_ | 2.43 | -1.45~ 6.31 | 0.22 | 1.34 | -3.57~ 6.26 | 0.59 |
| Ln SPINA-GT | -0.34 | -4.07~ 3.39 | 0.86 | -0.29 | -5.00~ 4.42 | 0.90 |
| Ln SPINA-GD | 7.92 | -2.91~ 18.75 | 0.15 | 11.31 | -2.33~ 24.95 | 0.10 |

TT_4_RI and TT_3_RI were ln-transformed for normal distribution before linear regression analysis.

Model 2: age, sex, body mass index, waist circumference, heart rate, diabetes, hypertension, dyslipidemia, TPOAb positive and TgAb positive were adjusted.

FT_3_: free triiodothyronine; FT_4_: free thyroxine; TSH: thyroid-stimulating hormone; FT_3_/FT_4_, FT_3_ to FT_4_ ratio, TSHI: TSH index; TT_4_RI: thyrotroph T_4_ resistance index; PTFQI_FT4_: Parametric Thyroid Feedback Quantile-based Index calculated by FT_4_; PTFQI_FT3_: Parametric Thyroid Feedback Quantile-based Index calculated by FT_3_; TT_3_RI: thyrotroph T_3_ resistance index; SPINA-GT: the secretory capacity of the thyroid gland; SPINA-GD: the sum activity of peripheral deiodinases; TPOAb: thyroid peroxidase antibody; TgAb: thyroglobulin antibody; eGFR _CKD-EPI_: estimated glomerular filtration rate based on CKD-EPI equation; eGFR _Cr-CysC_: eGFR based on serum creatinine and cystatin-C

**Supplementary table 2. Association of sensitivity of thyroid hormone indices with reduced renal function by logistic regression stratified by age**

| **(+1SD)** | **Younger than 65 years** | | | **Older than 65 years** | | | ***P* _for interaction_** |
| --- | --- | --- | --- | --- | --- | --- | --- |
|  | **OR** | **95%CI** | ***P*** | **OR** | **95%CI** | ***P*** |  |
| **eGFR _CKD-EPI_ <90 mL/min/1.73m^2^** | | | | | | |  |
| FT_3_/FT_4_ | 0.82 | 0.69~0.97 | 0.02 | 0.77 | 0.55~1.07 | 0.12 | 0.23 |
| TSHI | 1.41 | 1.19~1.67 | <0.001 | 1.08 | 0.81~1.44 | 0.60 | 0.04 |
| TT_4_RI | 1.39 | 1.19~1.62 | <0.001 | 1.08 | 0.79~1.46 | 0.64 | 0.07 |
| PTFQI_FT4_ | 1.34 | 1.13~1.57 | <0.001 | 1.12 | 0.82~1.55 | 0.47 | 0.32 |
| TT_3_RI | 1.28 | 1.10~1.49 | 0.001 | 0.99 | 0.74~1.32 | 0.93 | 0.03 |
| PTFQI_FT3_ | 1.13 | 0.96~1.33 | 0.14 | 0.90 | 0.67~1.21 | 0.48 | 0.05 |
| SPINA-GT | 0.76 | 0.62~0.92 | 0.006 | 1.03 | 0.81~1.31 | 0.79 | 0.01 |
| SPINA-GD | 0.82 | 0.69~0.97 | 0.02 | 0.77 | 0.55~1.07 | 0.12 | 0.23 |
| **eGFR _Cr-CysC_ <90 mL/min/1.73m^2^** | | | | | | |  |
| FT_3_/FT_4_ | 0.85 | 0.72~1.00 | 0.04 | 0.80 | 0.57~1.10 | 0.17 | 0.31 |
| TSHI | 1.32 | 1.12~1.56 | 0.001 | 1.08 | 0.81~1.43 | 0.61 | 0.08 |
| TT_4_RI | 1.32 | 1.14~1.53 | <0.001 | 1.14 | 0.84~1.54 | 0.40 | 0.17 |
| PTFQI_FT4_ | 1.29 | 1.10~1.50 | 0.002 | 1.10 | 0.80~1.51 | 0.57 | 0.28 |
| TT_3_RI | 1.24 | 1.07~1.43 | 0.004 | 1.07 | 0.80~1.42 | 0.65 | 0.09 |
| PTFQI_FT3_ | 1.11 | 0.95~1.30 | 0.19 | 0.91 | 0.68~1.22 | 0.53 | 0.07 |
| SPINA-GT | 0.88 | 0.74~1.04 | 0.14 | 1.01 | 0.80~1.28 | 0.95 | 0.16 |
| SPINA-GD | 0.85 | 0.72~1.00 | 0.04 | 0.80 | 0.57~1.10 | 0.17 | 0.31 |

ORs are estimated with generalized logistic regression models for the increase of thyroid parameters (1 SD).

Model 2: age, sex, body mass index, waist circumference, heart rate, diabetes, hypertension, dyslipidemia, TPOAb positive and TgAb positive were adjusted.

FT_3_: free triiodothyronine; FT_4_: free thyroxine; TSH: thyroid-stimulating hormone; FT_3_/FT_4_, FT_3_ to FT_4_ ratio, TSHI: TSH index; TT_4_RI: thyrotroph T_4_ resistance index; PTFQI_FT4_: Parametric Thyroid Feedback Quantile-based Index calculated by FT_4_; PTFQI_FT3_: Parametric Thyroid Feedback Quantile-based Index calculated by FT_3_; TT_3_RI: thyrotroph T_3_ resistance index; SPINA-GT: the secretory capacity of the thyroid gland; SPINA-GD: the sum activity of peripheral deiodinases; TPOAb: thyroid peroxidase antibody; TgAb: thyroglobulin antibody; eGFR _CKD-EPI_: estimated glomerular filtration rate based on CKD-EPI equation; eGFR _Cr-CysC_: eGFR based on serum creatinine and cystatin-C

**Supplementary table 3. Association of sensitivity of thyroid hormone indices with eGFR by linear regression classified by renal function**

|  | **eGFR _CKD-EPI_** | | | **eGFR _Cr-CysC_** | | |
| --- | --- | --- | --- | --- | --- | --- |
|  | **β** | **95%CI** | ***P*** | **β** | **95%CI** | ***P*** |
| **Normal renal function** | | | | | | |
| FT_3_/FT_4_ | 17.80 | 12.69~22.90 | <0.001 | 13.61 | 6.01~21.20 | <0.001 |
| TSHI | -1.30 | -1.79~ -0.81 | <0.001 | -1.75 | -2.47~ -1.02 | <0.001 |
| Ln TT_4_RI | -1.16 | -1.67~ -0.66 | <0.001 | -1.52 | -2.26~ -0.77 | <0.001 |
| PTFQI_FT4_ | -2.13 | -2.78~ -1.49 | <0.001 | -3.05 | -4.00~ -2.10 | <0.001 |
| Ln TT_3_RI | -0.63 | -1.13~ -0.14 | 0.01 | -1.09 | -1.82~ -0.35 | 0.004 |
| PTFQI_FT3_ | -0.07 | -0.72~ 0.58 | 0.84 | -1.66 | -2.62~ -0.70 | 0.001 |
| Ln SPINA-GT | 0.54 | -0.18~1.25 | 0.14 | 0.45 | -0.60~1.51 | 0.40 |
| Ln SPINA-GD | 5.74 | 4.03~7.45 | <0.001 | 4.45 | 1.90~7.01 | 0.001 |
| **Reduced renal function** | | | | | | |
| FT_3_/FT_4_ | 12.58 | -19.85~45.01 | 0.45 | 28.37 | -1.70~58.43 | 0.06 |
| TSHI | -0.38 | -3.13~ 2.37 | 0.79 | -1.44 | -4.05~ 1.18 | 0.28 |
| Ln TT_4_RI | -0.61 | -3.42~ 2.19 | 0.67 | -1.62 | -4.28~ 1.04 | 0.23 |
| PTFQI_FT4_ | 0.79 | -3.02~ 4.60 | 0.68 | -1.29 | -4.99~ 2.41 | 0.49 |
| Ln TT_3_RI | -0.31 | -3.11~ 2.49 | 0.83 | -0.89 | -3.48~ 1.71 | 0.50 |
| PTFQI_FT3_ | 2.17 | -1.71~ 6.06 | 0.27 | 1.60 | -2.02~ 5.22 | 0.39 |
| Ln SPINA-GT | 1.44 | -2.51~5.40 | 0.47 | 2.19 | -1.57~5.95 | 0.25 |
| Ln SPINA-GD | 3.94 | -6.21~14.08 | 0.45 | 8.68 | -0.74~18.09 | 0.07 |

TT_4_RI and TT_3_RI were ln-transformed for normal distribution before linear regression analysis.

Model 2: age, sex, body mass index, waist circumference, heart rate, diabetes, hypertension, dyslipidemia, TPOAb positive and TgAb positive were adjusted.

FT_3_: free triiodothyronine; FT_4_: free thyroxine; TSH: thyroid-stimulating hormone; FT_3_/FT_4_, FT_3_ to FT_4_ ratio, TSHI: TSH index; TT_4_RI: thyrotroph T_4_ resistance index; PTFQI_FT4_: Parametric Thyroid Feedback Quantile-based Index calculated by FT_4_; PTFQI_FT3_: Parametric Thyroid Feedback Quantile-based Index calculated by FT_3_; TT_3_RI: thyrotroph T_3_ resistance index; SPINA-GT: the secretory capacity of the thyroid gland; SPINA-GD: the sum activity of peripheral deiodinases; TPOAb: thyroid peroxidase antibody; TgAb: thyroglobulin antibody; eGFR _CKD-EPI_: estimated glomerular filtration rate based on CKD-EPI equation; eGFR _Cr-CysC_: eGFR based on serum creatinine and cystatin-C

**Supplementary table 4. Association of sensitivity of thyroid hormone indices with eGFR by linear regression in non-diabetic and non-hypertensive subjects.**

|  | **eGFR _CKD-EPI_** | | | **eGFR _Cr-CysC_** | | |
| --- | --- | --- | --- | --- | --- | --- |
|  | **β** | **95%CI** | ***P*** | **β** | **95%CI** | ***P*** |
| **Non-diabetic subjects*** | | | | | | |
| FT_3_/FT_4_ | 24.86 | 18.03~ 31.68 | <0.001 | 26.50 | 16.50~35.60 | <0.001 |
| TSHI | -1.83 | -2.47~ -1.19 | <0.001 | -2.60 | -3.50~ -1.71 | <0.001 |
| Ln TT_4_RI | -1.73 | -2.39~ -1.06 | <0.001 | -2.33 | -3.25~ -1.40 | <0.001 |
| PTFQI_FT4_ | -2.63 | -3.49~ -1.77 | <0.001 | -4.17 | -5.36~ -2.98 | <0.001 |
| Ln TT_3_RI | -1.01 | -1.66~ -0.36 | <0.001 | -1.53 | -2.43~ -0.62 | <0.001 |
| PTFQI_FT3_ | 0.08 | -0.78~ 0.95 | 0.85 | -1.51 | -2.71~ -0.31 | 0.01 |
| Ln SPINA-GT | 1.18 | 0.24~2.11 | 0.01 | 0.96 | -0.35~2.27 | 0.15 |
| Ln SPINA-GD | 8.08 | 5.80~10.37 | <0.001 | 8.72 | 5.52~11.91 | <0.001 |
| **Non-hypertensive subjects+** | | | | | | |
| FT_3_/FT_4_ | 23.93 | 15.86~32.00 | <0.001 | 20.83 | 9.75~31.91 | <0.001 |
| TSHI | -1.97 | -2.73~ -1.21 | <0.001 | -2.52 | -3.55~ -1.48 | <0.001 |
| Ln TT_4_RI | -1.86 | -2.65~ -1.08 | <0.001 | -2.29 | -3.36~ -1.21 | <0.001 |
| PTFQI_FT4_ | -2.89 | -3.89~ -1.88 | <0.001 | -4.01 | -5.38~ -2.64 | <0.001 |
| Ln TT_3_RI | -1.18 | -1.96~ -0.41 | 0.003 | -1.69 | -2.75~ -0.63 | 0.002 |
| PTFQI_FT3_ | -0.33 | -1.37~ 0.70 | 0.53 | -2.00 | -3.41~ -0.59 | 0.005 |
| Ln SPINA-GT | 1.39 | 0.26~ 2.52 | 0.02 | 1.21 | -0.33~ 2.75 | 0.12 |
| Ln SPINA-GD | 7.93 | 5.19~ 10.67 | <0.001 | 6.81 | 3.04~ 10.57 | <0.001 |

TT_4_RI and TT_3_RI were ln-transformed for normal distribution before linear regression analysis.

* Age, sex, body mass index, waist circumference, heart rate, hypertension, dyslipidemia, TPOAb positive and TgAb positive were adjusted in this group.

+ Age, sex, body mass index, waist circumference, heart rate, diabetes, dyslipidemia, TPOAb positive and TgAb positive were adjusted in this group.

FT_3_: free triiodothyronine; FT_4_: free thyroxine; TSH: thyroid-stimulating hormone; FT_3_/FT_4_, FT_3_ to FT_4_ ratio, TSHI: TSH index; TT_4_RI: thyrotroph T_4_ resistance index; PTFQI_FT4_: Parametric Thyroid Feedback Quantile-based Index calculated by FT_4_; PTFQI_FT3_: Parametric Thyroid Feedback Quantile-based Index calculated by FT_3_; TT_3_RI: thyrotroph T_3_ resistance index; SPINA-GT: the secretory capacity of the thyroid gland; SPINA-GD: the sum activity of peripheral deiodinases; TPOAb: thyroid peroxidase antibody; TgAb: thyroglobulin antibody; eGFR _CKD-EPI_: estimated glomerular filtration rate based on CKD-EPI equation; eGFR _Cr-CysC_: eGFR based on serum creatinine and cystatin-C

**Supplementary table 5. Association of sensitivity of thyroid hormone indices with reduced renal function by logistic regression in non-diabetic and non-hypertensive subjects.**

| **(+1SD)** | **eGFR _CKD-EPI_ <90 mL/min/1.73m^2^** | | | **eGFR _Cr-CysC_ <90 mL/min/1.73m^2^** | | |
| --- | --- | --- | --- | --- | --- | --- |
|  | **OR** | **95%CI** | ***P*** | **OR** | **95%CI** | ***P*** |
| **Non-diabetic subjects*** | | | | | | |
| FT_3_/FT_4_ | 0.79 | 0.67~0.93 | 0.005 | 0.83 | 0.71~0.97 | 0.02 |
| TSHI | 1.30 | 1.11~1.52 | 0.001 | 1.28 | 1.10~1.49 | 0.002 |
| TT_4_RI | 1.30 | 1.12~1.51 | <0.001 | 1.29 | 1.12~1.49 | <0.001 |
| PTFQI_FT4_ | 1.29 | 1.11~1.51 | 0.001 | 1.29 | 1.10~1.50 | 0.001 |
| TT_3_RI | 1.19 | 1.03~1.38 | 0.02 | 1.20 | 1.04~1.38 | 0.01 |
| PTFQI_FT3_ | 1.06 | 0.91~1.23 | 0.48 | 1.08 | 0.93~1.26 | 0.30 |
| SPINA-GT | 0.89 | 0.77~1.04 | 0.14 | 0.93 | 0.81~1.08 | 0.33 |
| SPINA-GD | 0.79 | 0.67~0.93 | 0.005 | 0.83 | 0.71~0.97 | 0.02 |
| **Non-hypertensive subjects+** | | | | | | |
| FT_3_/FT_4_ | 0.77 | 0.62~0.96 | 0.02 | 0.90 | 0.73~1.12 | 0.34 |
| TSHI | 1.22 | 0.99~1.51 | 0.06 | 1.21 | 0.98~1.50 | 0.08 |
| TT_4_RI | 1.22 | 0.99~1.49 | 0.06 | 1.25 | 1.02~1.53 | 0.03 |
| PTFQI_FT4_ | 1.22 | 0.99~1.49 | 0.06 | 1.20 | 0.98~1.49 | 0.08 |
| TT_3_RI | 1.11 | 0.91~1.36 | 0.29 | 1.19 | 0.98~1.46 | 0.09 |
| PTFQI_FT3_ | 0.97 | 0.79~1.20 | 0.80 | 1.09 | 0.88~1.36 | 0.41 |
| SPINA-GT | 0.87 | 0.70~1.09 | 0.24 | 0.93 | 0.75~1.16 | 0.51 |
| SPINA-GD | 0.77 | 0.62~0.96 | 0.02 | 0.90 | 0.73~1.12 | 0.35 |

ORs are estimated with generalized logistic regression models for the increase of thyroid parameters (1 SD).

*Age, sex, body mass index, waist circumference, heart rate, hypertension, dyslipidemia, TPOAb positive and TgAb positive were adjusted in this group.

+ Age, sex, body mass index, waist circumference, heart rate, diabetes, dyslipidemia, TPOAb positive and TgAb positive were adjusted in this group.

FT_3_: free triiodothyronine; FT_4_: free thyroxine; TSH: thyroid-stimulating hormone; FT_3_/FT_4_, FT_3_ to FT_4_ ratio, TSHI: TSH index; TT_4_RI: thyrotroph T_4_ resistance index; PTFQI_FT4_: Parametric Thyroid Feedback Quantile-based Index calculated by FT_4_; PTFQI_FT3_: Parametric Thyroid Feedback Quantile-based Index calculated by FT_3_; TT_3_RI: thyrotroph T_3_ resistance index; SPINA-GT: the secretory capacity of the thyroid gland; SPINA-GD: the sum activity of peripheral deiodinases; TPOAb: thyroid peroxidase antibody; TgAb: thyroglobulin antibody; eGFR _CKD-EPI_: estimated glomerular filtration rate based on CKD-EPI equation; eGFR _Cr-CysC_: eGFR based on serum creatinine and cystatin-C

**Supplementary figure 1. Partial regression plots of association between sensitivity of thyroid hormone indices and eGFR _CKD-EPI._**

A. **FT_3_/FT_4_**


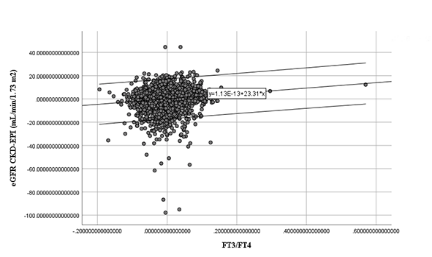


B. **TSHI**


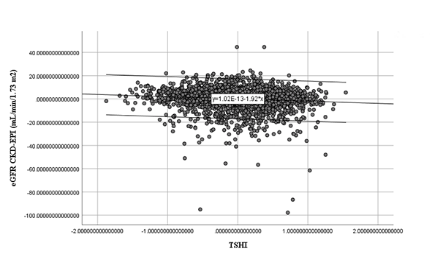


C. **Ln TT_4_RI**


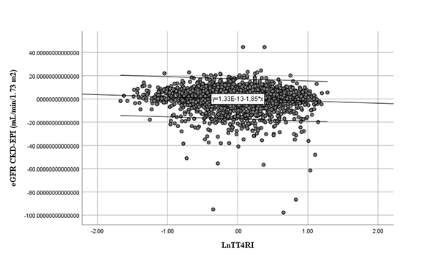


D. **PTFQI_FT4_**


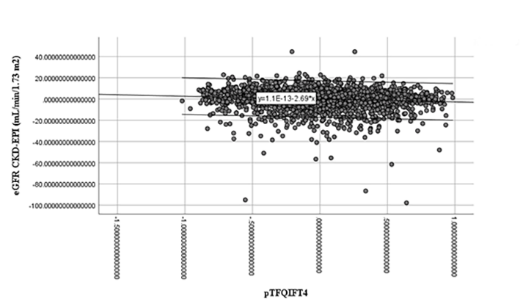


**E. Ln SPINA-GT**

**
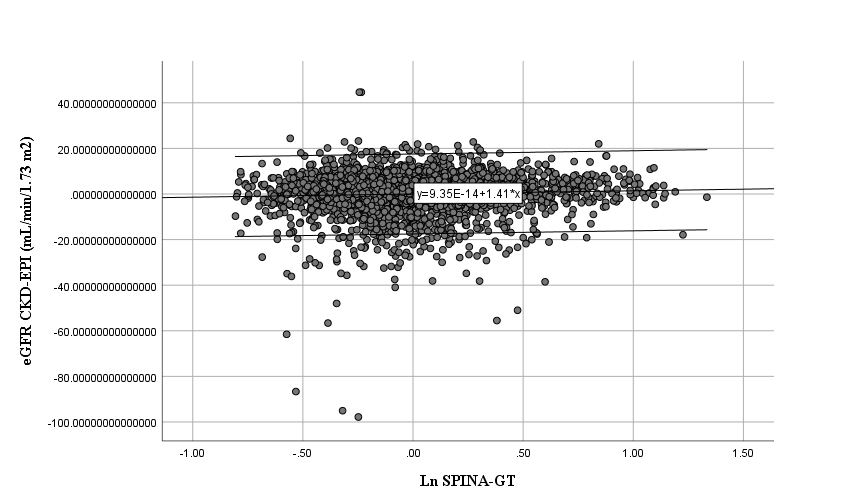
**

**F. Ln SPINA-GD**

**
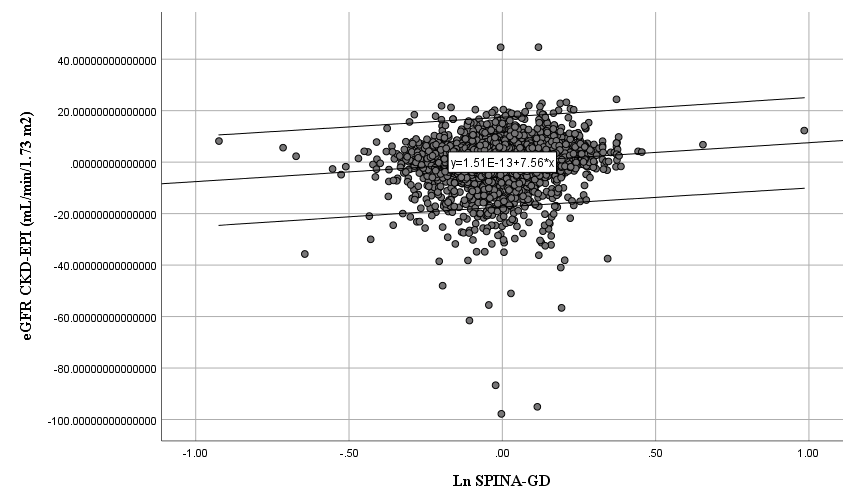
**

TT_4_RI, SPINA-GT and SPINA-GD were ln-transformed for normal distribution before linear regression analysis.

Model 2: age, sex, body mass index, waist circumference, heart rate, diabetes, hypertension, dyslipidemia, TPOAb positive and TgAb positive were adjusted.

FT_3_: free triiodothyronine; FT_4_: free thyroxine; TSH: thyroid-stimulating hormone; FT_3_/FT_4_, FT_3_ to FT_4_ ratio, TSHI: TSH index; TT_4_RI: thyrotroph T_4_ resistance index; PTFQI_FT4_: Parametric Thyroid Feedback Quantile-based Index calculated by FT_4_; SPINA-GT: the secretory capacity of the thyroid gland; SPINA-GD: the sum activity of peripheral deiodinases; eGFR _CKD-EPI_: estimated glomerular filtration rate based on CKD-EPI equation

1. This author’s affiliation name changed during peer-review process. The new affiliation is as follows: Department of Endocrinology and Metabolism, Institute of Endocrinology, NHC Key Laboratory of Diagnosis and Treatment of Thyroid Diseases, The First Affiliated Hospital of China Medical University, 155 Nanjing Bei Street, Shenyang, Liaoning 110001, P.R. China. [↑](#footnote-ref-1)
